# Frequency of Vitamin D deficiency in pregnant diabetics at Baskent University Hospital, Istanbul

**DOI:** 10.12669/pjms.291.2896

**Published:** 2013

**Authors:** Hulya Parildar, Asli Dogruk Unal, Guldeniz Aksan Desteli, Ozlem Cigerli, Nilgun Guvener Demirag

**Affiliations:** 1 Hulya Parildar, MD, Department of Family Medicine, Baskent University, Istanbul Hospital, Mahir Iz caddesi No: 53 Altunizade/Uskudar,Istanbul, Turkey.; 2 Asli Dogruk Unal, MD, Department of Endocrinology and Metabolism, Baskent University, Istanbul Hospital, Oymaci Sokak No: 7 Altunizade/Uskudar, Istanbul, Turkey.; 3 Guldeniz Aksan Desteli, MD, Department of Gynecology and Obstetrics, Baskent University, Istanbul Hospital, Oymaci Sokak No:7 Altunizade/Uskudar Istanbul, Turkey.; 4 Ozlem Cigerli, MD, Department of Family Medicine, Baskent University, Istanbul Hospital, Mahir Iz caddesi No: 53 Altunizade/Uskudar,Istanbul, Turkey.; 5 Nilgun Guvener Demirag, MD, Department of Endocrinology and Metabolism, Baskent University, Istanbul Hospital, Oymaci Sokak No: 7 Altunizade/Uskudar, Istanbul, Turkey.

**Keywords:** Vitamin D, Gestational diabetes, Nutrition in pregnancy

## Abstract

***Objective:*** To find out the frequency of vitamin D deficiency and its relation with glucose parameters and the incidence of gestational diabetes (GDM).

***Methodology:*** Gestational diabetes was diagnosed with 75 gram oral glucose tolerance test. Forty-four pregnant women diagnosed with GDM and 78 non-GDM pregnant women were enrolled as case and control group, respectively in this descriptive study. Vitamin D status was classified as deficiency at ≤20 ng/ml for serum 25(OH)D concentrations.

***Results: ***The mean ages were 33.4±5.2 (18-44) years and 29.7±4.1 (21-39) years, mean BMI was 30.6±5.9 kg/m² (19.5-46.1) and 25.9±4.4 kg/m2 (16.5-38) in case and control groups, respectively. The frequency of GDM was found 9.38%. The mean serum vitamin D levels in GDM group were significantly lower than in non-GDM subjects (p=0.07). A total of 56.8% of GDM patients were compared with 35.8% of control group which had Vitamin D deficiency and the difference was significant (p= 0.02). There was no significant association between vitamin D levels and fasting glucose, insulin and HbA1c. Vitamin D levels were inversely correlated with clothing style, parathyroid hormone levels, dental problems and muscle cramps.

***Conclusions:*** The association of maternal Vitamin D status with the markers of glucose metabolism in pregnancy needs prospective studies.

## Introduction

 Gestational diabetes mellitus (GDM) is defined as glucose intolerance that is first diagnosed during pregnancy resulting from pregnancy associated insulin resistance and impaired insulin secretion.^1^ Women with GDM are at high risk for gestational or delivery problems and developing diabetes after the delivery.^1^

 The Vitamin D deficiency is also prevalant all over the world and became an important topic of the studies as its potential negative effects were reported on glucose metabolism and associated diseases.^2^ It was shown in many studies that the secretion of placental hormones have been regulated and the inflammatory cytokines that stimulate preeclampsia and premature labor are inhibited by 1.25(OH)_2_D.^3^^,^^4^ But the studies investigating the impact of Vitamin D status on glucose homeostasis during pregnancy and on the development of gestational diabetes mellitus are limited and the findings are inconsistent.^5^^-^^10^

 We aimed to investigate the frequency of Vitamin D deficiency in pregnant women with gestational diabetes and the relation of Vitamin D levels with the glucose parameters in the presence of gestational diabetes.

## Methodology

 A total of 44 pregnant women with gestational diabetes and 78 healthy non-GDM pregnant women who presented at Gynecology and Obstetric Department of Baskent University Hospital Istanbul between 2009 and 2011 were included in this cross-sectional, descriptive and controlled trial. The diagnosis of gestational diabetes was determined by the guidelines proposed by the International Association of Diabetes and Pregnancy Study Groups (IADPSG) with a 75-gr 2 hour oral glucose tolerance test at 24-32 weeks’ gestation. GDM is diagnosed when the fasting serum glucose is 92 mg/dL or greater, or the 1-hour value is 180 mg/dL or greater or when the 2-hour value is 153 mg/dL or greater. The subjects were divided into two groups according to the presence of gestational diabetes as case (group 1) and control (group 2).

 Serum fasting insulin, 25 (OH) D, creatinine, calcium (Ca), phosphorus (P), magnesium (Mg), alkaline phosphatase (ALP), parathyroid hormone (PTH), thyroid-stimulating hormone (sTSH), albumin, HbA1c, liver transaminases (AST and ALT) were also measured and the body mass indexes (BMI) were calculated in all groups. Serum 25 (OH) D levels ≤20 ng/mL are accepted as the cutoff level for vitamin D deficiency since this level was the highest level to maintain the serum PTH levels below 45 pg/ml. Pregnant women with history of pregestational diabetes, multiple pregnancies, fetal abnormality, chronic renal or liver failure and history of consumption of calcium supplements or vitamin D were excluded from the study. All subjects gave written informed consent and the study was approved by the local ethics committee (KAO9/355).

 All patients were asked about their consumption of prenatal vitamin/food supplements, dairy products/fish and any drugs that may alter glucose metabolism and antihypertensive medication, family history of diabetes and obesity, the period of daily physical activity and sun exposure.

 These collected maternal fasting plasma samples were kept frozen at −20°C. Serum glucose was measured by using an enzymatic in vitro test. 25-hydroxy vitamin D3 was analyzed by a human ELISA kit (Immuno Diagnostic System, UK), with interassay coefficient of variation (CV) of 2.6% intraassay CV of 2.3%.

 Serum Ca, P, Mg, (ALP) and glucose levels were measured using the enzymatic colorimetric method  (Roche Integra 800), while  serum 25 hydroxy vitamin D 25(OH) D levels were measured during the fall and the winter with a chemiluminescent immunassay method (CMIA) (Architect i1000 system, Abbott, USA) Normal ranges were between (NA): 15.7-60.3ng/ml (summer), 8.8-46.3ng/ml (winter). The intra-assay variation ranged from 2.6-4%. Insulin levels were analysed with CMIA (Architect i1000 system, Abbott, USA), NA: 2.6-24.9 μU/ml. Intra-assay coefficients of variation were 2.3-4.2%. Serum PTH levels were measured with electrochemiluminescent immunassay method (ECLIA) (Architect i2000 system, Abbott, USA), NA:15-68pg/ml. Intra-assay coefficients of variation was 3-6.5%.

 Serum calcium (Ca) and phosphorus (P) levels were measured with enzymatic colorimetric assay (C8000 system, Abbott, USA). Intra and inter-assay coefficients of variation were 0.5-0.6%, and 0.5-0.3% for Ca; 0.5-0.5% and 0.3-0.6% for P. Serum glucose measured with enzymatic colorimetric assay (C8000 system, Abbott, USA) intra and interassay coefficients of variation were 1.98-0.65% and 0.84-0.93% respectively. HbA1c was detected with turbidimetric assay method (C4000, Architect cSystem, Abbott, USA) Intra and interassay coefficients of variation were 0.88-0.77% and 1.88-1.45%, respectively.

 Statistical analysis was performed using SPSS 16.0 for Windows. Student T test, Fisher’s exact test and Spearman corelation test were used for the comparisons of categorical variables and correlations between two variables. Statistical significance was set at p value of less than 0.05.

## Results

 A total of 413 pregnant women were screened during the study period and 44 pregnant women were diagnosed with GDM. Seventy-eight non GDM pregnant women were enrolled as control group. All subjects were matched in their multivitamin use, magnesium levels, family history of diabetes and obesity, sun exposure and daily physical activities. Sociodemographic characteristics and metabolic parameters of the study groups are shown in Table-I. There was a statistically significant difference regarding the prevalance of Vitamin D deficiency between case and control groups (x²=5.01, p=0.02) (Table-II).

**Table-I:** Comparison of case and control groups in terms of age, BMI, HbA1c and serum Vitamin D and fasting insulin levels.

	*Control* *(n=78)*	*Case* *(n=44)*	*p*
Age (year)	29.9±4.1(min=21, max=39)	33.4±5.2(min=18, maks=44)	<0.001
BMI (kg/m²)	25.9±4.4(min=16.5, max=38)	30.6±5.9(min=19.5, max=46.1)	<0.001
Vitamin D (ng/ml)	22.9±10.0(min=6.6, max=73)	19.5±9.3(min=4, max=39.3)	0.07
HbA1c (%)	4.7±0.6(min=4, max=5.7)	5.4±0.8(min=4.2, max=8.8)	0.004
Insulin (µu/ml)	17.4±13.8(min=5.2, max=51.7)	14.3±9.6 3(min=3.7, max=42.6)	0.5
Fasting serum glucose (mg/dl)	83.4±9.2(min=68, max=91)	95.0±13.8(min=59, max=126)	<0.01

**Table-II:** Vitamin D levels in case and control groups (x²=5.01, p=0.02).

*Vitamin D levels and case-control crosstabulation*		*Control*	*Case*	*Total*	*p*
Vitamin D	<20ng/ml	28	25	53	0,020
	>20ng/ml	50	19	69	
	Total	78	44	122	

**Table-III:** The association between Vitamin D levels and metabolic parameters in case group.

	*Vitamin D <20ng/ml*	*Vitamin D >20ng/ml*	*p*
HbA1c (%)	5.5±0.6 (min=4.8, max=7.1)	5.3±1.1 (min=4.2, max=8.8)	0,1
Insulin (µu/ml)	16.7±10.8 (min=4.6, max=42.5)	10.8±5.3 (min=3.7, max=21.2)	0,2

**Fig.1:**
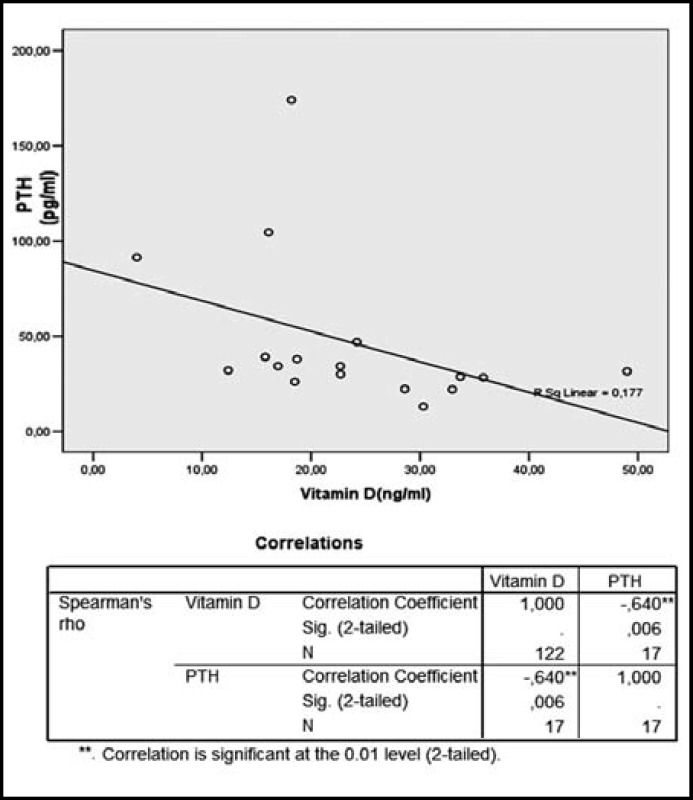
The correlation between serum Vitamin D and PTH levels

 There was a negative correlation between serum 25 (OH) D and parathyroid hormon levels (r=-0.7, p=0.003) (Fig.1). There was no significant difference between serum Vitamin D and serum fasting glucose, insulin and HbA1c levels (Table-III).

 Family history of diabetes and obesity was 73.3% (n=34) and 45.5% (n=20), respectively in pregnants with GDM. These rates were 77.8% (n=35) and 46.7% (n=21) respectively in control. Only 12% of group 1 and 11.5% of group 2 were engaged in regular physical activity of 30 min/day duration, the others were not doing any type of exercise. Dairy products consumed per day were approximately 2.4 portions in all groups. 

**Fig.2:**
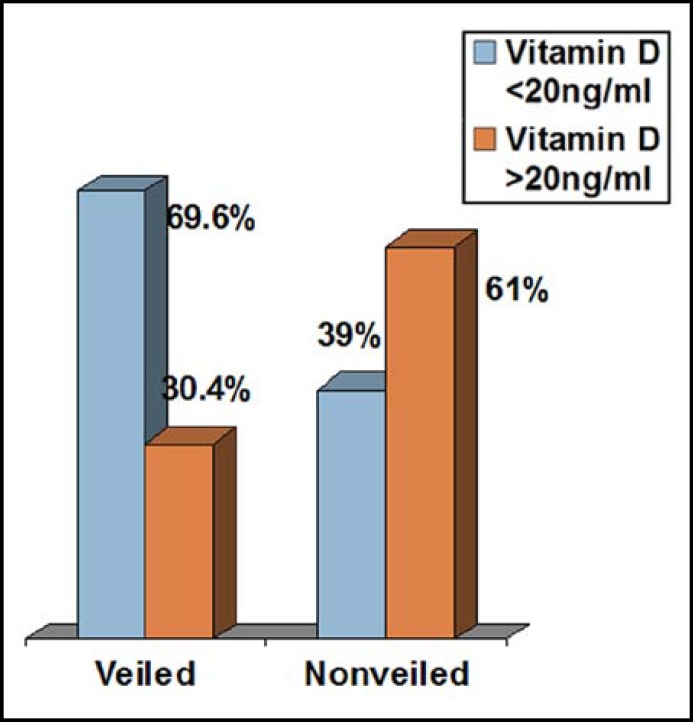
Serum Vitamin D levels in veiled and nonveiled pregnant

 The mean Vitamin D levels of veiled pregnant women were significantly lower compared to nonveiled (14.3±8.2, 23.2±8.3, respectively p=0.001) in pregnant women (Fig.2).

**Fig.3:**
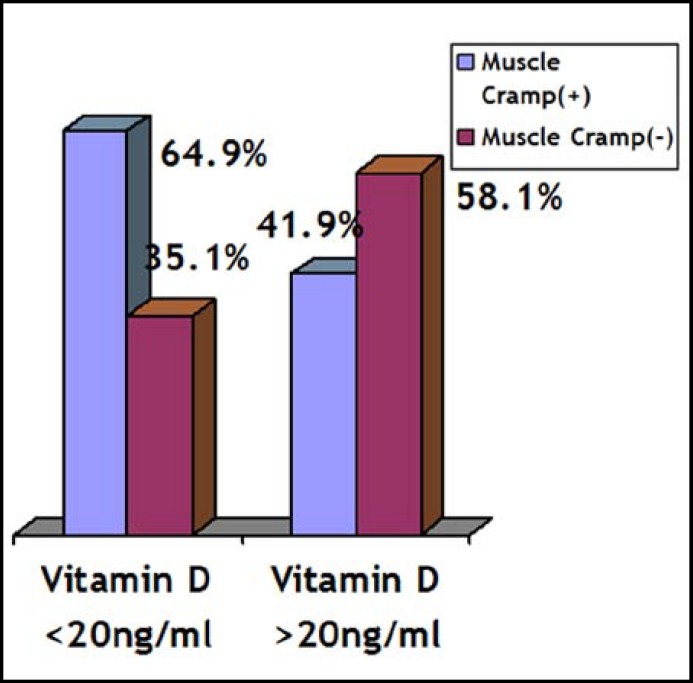
Vitamin D levels and the frequency of muscle cramps (x²=3.57, p=0.05).

**Fig.4:**
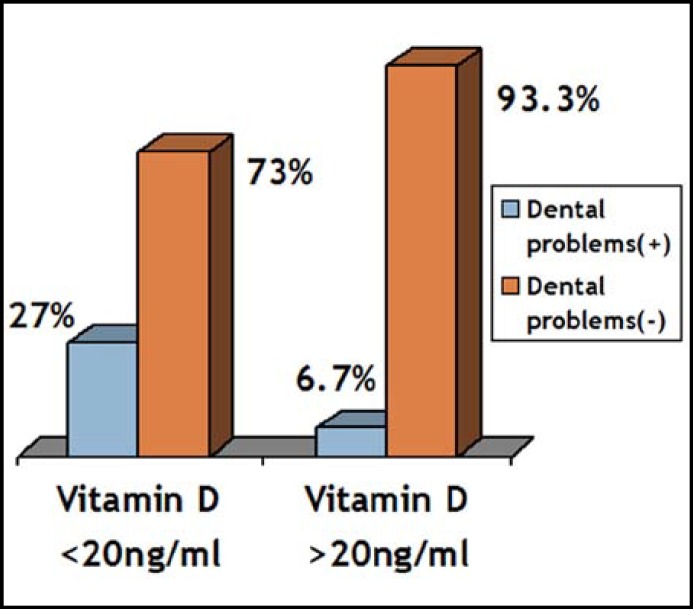
Vitamin D levels and the frequency of dental problems (x²=4.67, p=0.03).

 The main symptoms in Vitamin D deficient pregnant women were dental problems, muscle cramps, weakness, soreness and sweating. Dental problems and muscle cramps were seen more frequent in Vitamin D deficient pregnant women compared to non-vitamin D deficient pregnant women (Fig. 3 and 4).

## Discussion

 The frequency of GDM was found among 9.38% women in our study. This rate was consistent with the literature regarding the increasing gestational diabetes prevalance in the world.^1^

 Our study provides data indicating that maternal Vitamin D deficiency (serum 25-[OH] D <20 ng/mL) is prevalent among our pregnant population. This evidence is consistent with low vitamin D levels in either pregnant or non-pregnant individuals in our country as in the rest of the world.^11^^,^^12^ However we did not find significant association of low levels of serum 25 (OH) D with elevated risk for GDM even after adjustment for conventional risk factors for diabetes. Data relating vitamin D status to GDM risk are inconsistent and limited. In one cross-sectional study, serum 25(OH) D levels during 24-28 weeks of gestation were lower in GDM women than in nondiabetic pregnant women.^9^ In another study, maternal serum 25 (OH) D concentrations measured at the time of GDM screening test were significantly and inversely associated with fasting glucose, although the association of Vitamin D with GDM risk was not statistically significant.^8^ Our findings are also consistent with a study in an Indian population, showing no significant association between 25 (OH) D concentrations (at 30 weeks of gestation) and GDM risk.^6^

 Deficiency of vitamin D was shown to be associated with fetal and brain growth deficiency and type 1 diabetes in children.^13^ Significantly reduced risk of type 1 diabetes mellitus development was found when high doses of vitamin D supplementation (up to 2000 IU/d) were given during infancy in a birth-cohort study.^14^

 Vitamin D deficiency was also associated with increased risk for pregnancy complications and serious health problems in the offspring.^5^^,^^15^^-^^17^ Higher risks of preeclampsia and cesarean section ratios were shown in pregnants with low levels of Vitamin D.^16^^,^^17^ Moreover supplementation of vitamin D has been demonstrated to decrease the risk of preeclampsia by 27% in a study.^18^

 There are studies showing that prenatal vitamins including 400 IU of Vitamin D are insufficient to supplement the low levels of 25(OH)D in pregnant women.^19^ In the present study, we did not find any significant difference in engaging physical activity between two groups. Outdoor physical activity, since correlated with sun exposure, could be a protective factor for insulin resistance, obesity and GDM.^20^^-^^22^ Vitamin D deficiency can result from a variety of causes including malabsorption problems, obesity, age, skin color, altitude, lack of Vitamin D in breast milk and in diet, the side effects of certain medications and inadequate exposure to sunlight. 

 Although intervention trials assessing the role of Vitamin D supplementation on glucose metabolism among pregnant an/or non-pregnant individuals have inconsistent results and, the dose of supplementation and the duration of follow up period were not standardised in these studies, there is a growing evidence for potential benefits of high-dose Vitamin D supplementation during pregnancy.^23^^-^^25^

 Our study has several limitations. First, serum 25 (OH) D concentrations determined in late trimester may not show the status of maternal vitamin D during the whole pregnancy period and hence to identify the association of GDM development and vitamin D status. Secondly, the BMI were not matched in our study. Longitudinal studies with serial measurements of maternal plasma 25(OH) D concentrations, and monitoring the development of gestational diabetes in BMI matched pregnant women in relation with these measurements are required to explore the pathophysiology.

## Conclusions

 Our study has contributed to data regarding the increasing prevalance of gestational diabetes and the growing evidence of Vitamin D deficiency in pregnant population. Although there is no consensus for routine Vitamin D screening in pregnancy, being alert about the Vitamin D deficiency and its symptoms and optimising when necessary by replacement and/or through taking lifestyle measures may be recommended to prevent the birth of Vitamin D-deficient infants from vitamin D-deficient mothers. The lifestyle measures can include increasing the sun exposure and outdoor physical activity which also contribute to weight control and thus improving glucose homeostasis and/or favourable outcomes in pregnancy. Besides the safety of Vitamin D supplementation has already been known. Further studies are needed to determine the optimal levels and doses of Vitamin D in pregnancy to reduce the health risks for both mother and fetus. Investigating and diagnosing the factors that may negatively affect the development of gestational diabetes as early as possible is very important.
